# Global practices in regard to implementation of preventive measures for leprosy

**DOI:** 10.1371/journal.pntd.0005399

**Published:** 2017-05-04

**Authors:** Laura Gillini, Erwin Cooreman, Tanya Wood, Venkata Rao Pemmaraju, Paul Saunderson

**Affiliations:** 1 Global Leprosy Program, World Health Organization, Regional Office for South-East Asia, New Delhi, India; 2 International Federation of Anti-Leprosy Associations (ILEP), Geneva, Switzerland; 3 American Leprosy Missions, Greenville, South Carolina, United States of America; Kwame Nkrumah University of Science and Technology, GHANA

## Introduction

The global incidence of leprosy is declining slowly, but it is not known why this is happening. Suggested reasons include the gradually increasing global standard of living, increasing coverage with Bacillus Calmette–Guérin (BCG) vaccination of infants over the last 90 years or so, and specific measures to find and treat patients with leprosy over the last 60 years or so. None of these has a large effect and there is interest in finding other ways to speed up the decline in incidence by developing more targeted measures to interrupt the transmission of leprosy in the community.

The number of patients on treatment for leprosy (the registered prevalence) has declined steadily over the last three decades, largely in parallel with the decreasing duration of treatment. The number of new cases detected each year has shown a much smaller, but variable decline in different countries [1], and is strongly influenced by policies relating to case finding. In fact, with the integration of health services, active case-finding activities have been reduced and it is likely that a significant number of incident cases are not being detected [2]. The continuing incidence of leprosy in children in approximately 100 countries suggests that transmission of the disease continues and indicates the need for a global strategy of prevention.

Rising living standards and the gradually improving coverage of BCG vaccine in infants are two general changes that may have exerted downward pressure on the incidence of leprosy, although the effect is difficult to quantify [3]. Treatment with powerful bactericidal drugs does not seem to have had a major impact on incidence [3], probably because most transmission occurs before initiating treatment.

The Global Leprosy Programme (GLP), based in the WHO Regional Office for South-East Asia (SEARO) in New Delhi, plans to issue new guidelines on the treatment and prevention of leprosy. To date, guidance on leprosy management has come from meeting reports, such as those of the Expert Committee or strategy documents and operational guidelines. Neither the 2010 Expert Committee Report [4], nor the 2009 Operational Guidelines [5], written as a guide to the implementation of the Global Leprosy Strategy 2011–2015 [6], recommended any specific preventive measures, although they did call for further research into the logistics and feasibility of prophylaxis for contacts of new cases.

Two possible strategies to reduce incidence are being studied. Firstly, if cases could be detected and started on treatment much earlier, there may be an effect on the transmission of *Mycobacterium leprae*, the causative organism of leprosy. Active case-finding, especially through (extended) contact surveys, may help, but this is still dependent on overt signs of clinical disease, which may already be too late. The use of diagnostic tests that could identify subclinical disease would be a better option, but this has proved to be a difficult technical challenge [7]. The second strategy involves post-exposure prophylaxis (PEP). It is assumed that some of the contacts of new cases will have been exposed to *M*. *leprae* and will be incubating leprosy. As the endemicity of leprosy declines, a gradually higher proportion of new cases is found amongst the contacts of known cases [8]. The use of an antibiotic to treat that very early infection (chemoprophylaxis) or a vaccine to stimulate an effective immune response (immunoprophylaxis) could stop the development of the disease and thus reduce incidence.

The evidence for and against different drug regimens for chemoprophylaxis has been reviewed. A definitive trial of single-dose rifampicin (SDR) in contacts of new cases in Bangladesh showed a protective effect of 56% for a period of two years, and this is the regimen that is currently most widely used for studies on prevention of leprosy [9]. It has the advantage of being cheap and simple. Worries that SDR may lead to increasing resistance to rifampicin in other bacteria, especially *M*. *tuberculosis*, have been allayed [10].

In 2007, the WHO set up clear administrative and technical mechanisms to regulate the issuance of official “WHO Guidelines.” Before being published, such guidelines need to go through a rigorous process of review, with a thorough search of available evidence, including information on the impact and feasibility of implementation at country level. Background information about the policies currently being implemented globally for prevention of leprosy must be collected as preparation for that process, in line with the 2016–2020 Global Leprosy Strategy, which calls for more evidence on leprosy transmission and a more holistic approach to leprosy prevention [11]. For that purpose, from March to May 2016, GLP carried out a survey to determine the extent of PEP implementation through chemotherapy or by immunization with BCG in countries around the world either as national policy or under research studies.

## Methods

A questionnaire accessible both online and through email was sent to the leprosy national focal points of all six WHO regional offices. Additionally, the questionnaire was circulated to the International Federation of Anti-Leprosy Associations (ILEP) for further distribution to their member non-governmental organizations (NGOs). The questionnaire enquired about current and/or past use of post-exposure chemoprophylaxis and/or BCG revaccination, as tools to prevent leprosy among contacts of leprosy cases under either national policies, service provision by NGOs and/or other institutions, or research studies. For countries where such measures have been or are in use, the questionnaire enquired about the target population, medication utilized and related doses, and/or vaccine used. If preventive tools were provided under a research study, the study protocol was requested and reviewed. Information on ongoing studies on post-exposure prophylaxis (PEP) have been collected from ILEP via interagency coordination.

## Results

Information relating to 39 countries was collected primarily through questionnaire submission. In light of the concurrent emergency response to the Zika outbreak threat, the WHO Regional Office for the Americas (AMRO) decided to fill in the questionnaire at the regional office after brief interviews with the country WHO technical staff devoted to supporting communicable diseases control (including for neglected tropical diseases); as a result, they were able to provide information in relation to practices in an additional 24 countries. A further three countries provided information through ILEP. Therefore, in total, this survey collected information with regard to practices in 66 countries globally, representing 30% of the 217 countries and territories targeted by the survey through the WHO regional offices, but more importantly, 95% of the total reported a global leprosy burden.

The information presented in Table 1 shows that the nationwide use of post-exposure chemoprophylaxis with SDR as a routine national policy is still very limited because it is implemented nationwide only in four countries, whereas some of the countries with the highest burden of the disease globally are implementing it as a subnational policy, usually in the most endemic districts.

**Table 1:** Summary of global practices in regard with use of post-exposure prophylaxis (PEP) using single-dose rifampicin (SDR) or other regimens by region.

Region	Countries using PEP-SDR as national policy (Year)	Countries using PEP-SDR as sub-national policy (Year)	Countries using PEP-SDR or other regimen under research studies (Year)
**AFR**	None	None	Democratic Republic of Congo 2015–2016 United Republic of Tanzania (2015–2018)
**AMR**	Cuba (2002) Peru (planned for end of 2016)	Brazil (planned for end of 2016)	None
**EMR**	Morocco (2014)	None	None
**SEAR**		India (2016) Indonesia (2014) Nepal (2015)	Myanmar (2015–2018) Sri Lanka (2015–2018)
**WPR**	Samoa (2016)	None	Cambodia (2016–2017)

AFR, WHO African Region; AMR, WHO Americas Region; EMR, WHO Eastern Mediterranean Region; PEP, post-exposure prophylaxis; SDR, single-dose rifampicin; SEAR, WHO South-East Asia Region; WPR, WHO Western Pacific Region.

The use of SDR for contacts is currently being implemented under a three-year, multi-country research study known as the Leprosy Post-Exposure Prophylaxis (LPEP), with the support of Novartis Foundation, Netherlands Leprosy Relief, American Leprosy Missions, German Leprosy, Tuberculosis Relief Association, and Fairmed in the following countries since 2015: India, Indonesia, Nepal, Myanmar, Sri Lanka, and United Republic of Tanzania. The WHO African Region, in collaboration with Damien Foundation and the National Leprosy Programme of the Democratic Republic of the Congo, is studying the feasibility and the long-term effect of the use of one month multibacillary multidrug therapy (MB-MDT) for household contacts of leprosy patients over a two-year period. In Cambodia, with the support of Novartis Foundation, the programme started a research study in 2016, which provides PEP (SDR) to contacts, including contacts of index cases from previous years; because the number of index cases is low, this study uses a small mobile team to provide SDR district by district, and thus covers the whole country without the need to train a large number of health staff.

The use of BCG vaccination as a tool for leprosy prevention, is shown in Table 2.

**Table 2:** Summary of global practices in regard with use of immunoprophylaxis for leprosy by region.

Region	Countries using BCG vaccination or revaccination of leprosy contacts as national policy (year)	Countries using BCG/other vaccine for leprosy contacts under research studies (Year)
**AFR**	None	None
**AMR**	Brazil (1987), Colombia (2000), Peru (planned for end of 2016)	None
**EMR**	None	None
**SEAR**	None	Bangladesh (2016) India (*Mycobacterium indicum pranii* vaccine, planned for the beginning of 2017)
**WPR**	Australia (2008)	None

AFR, WHO African Region; AMR, WHO Americas Region; BCG, Bacillus Calmette–Guérin; EMR, WHO Eastern Mediterranean Region; SEAR, WHO South-East Asia Region; WPR, WHO Western Pacific Region.

In northern Bangladesh, the so-called Maltalep trial examines the safety and efficacy of BCG as a PEP in household contacts, and the additive effect of SDR given two months later to the same contacts [12]. The safety concern is that a small proportion of contacts (approximately 0.4%) develop signs of clinical leprosy within 12 weeks of receiving BCG [13]; this is manageable on a small scale but may be a problem with more widespread use.

## Discussion

The routine use of BCG as a preventive tool for leprosy either on its own or in parallel with rifampicin chemoprophylaxis seems to be extremely limited globally, whereas the use of SDR as a preventive tool is becoming more frequent, even if in the majority of cases this is happening under research studies and/or pilot implementation research projects. For each preventive tool either on its own or in combination, there is published evidence of efficacy [14, 15]. While Brazil has officially sanctioned the use of BCG in contacts of new leprosy cases for three decades, very little is known about its implementation. Indonesia, Nepal, and India are planning a more widespread launch of chemoprophylaxis sub-nationally (in high endemic districts only) in 2016, but they are still in the phase of defining their national protocols and identifying procurement and distribution systems for rifampicin to be used by the leprosy programme. Routine implementation seems to have been put in place only in countries with a very low burden, such as Cuba (since 2002), Morocco (since 2014), following a pilot project run in 2013, and Samoa (since 2015). This scenario might change after the completion of the Novartis Foundation funded studies on the feasibility of the implementation of this measure and in consideration with the present start of pilot implementation projects in Brazil.

The main limitation of the survey is the partial response from countries, although the most endemic countries responded. Another limitation is the “WHO reported” information from AMRO and the possible implementation of preventive tools by NGOs that are not ILEP affiliated because NGOs often represent a significant portion of leprosy services.

Leprosy control programmes throughout the world will benefit from the availability of official WHO guidelines in regard to preventive tools for leprosy, and, therefore, the publication of the results of currently ongoing research studies and pilot projects is strongly encouraged.
